# Self-reported knowledge of tetrahydrocannabinol and cannabidiol concentration in cannabis products among cancer patients and survivors

**DOI:** 10.1007/s00520-024-08374-w

**Published:** 2024-03-05

**Authors:** Michelle Goulette, Nicolas J. Schlienz, Amy A. Case, Eric Hansen, Cheryl Rivard, Rebecca L. Ashare, Maciej L. Goniewicz, Maansi Bansal-Travers, Andrew Hyland, Danielle M. Smith

**Affiliations:** 1 Department of Health Behavior, Roswell Park Comprehensive Cancer Center, Elm & Carlton Streets, Buffalo, NY 14263 USA; 2 State University of New York at Buffalo, Buffalo, NY USA

**Keywords:** Cannabis, Tetrahydrocannabinol, Cannabidiol, Cancer

## Abstract

**Purpose:**

Cannabis use may introduce risks and/or benefits among people living with cancer, depending on product type, composition, and nature of its use. Patient knowledge of tetrahydrocannabinol (THC) or cannabidiol (CBD) concentration could provide information for providers about cannabis use during and after treatment that may aide in risk and benefit assessments. This study aimed to examine knowledge of THC or CBD concentration among patients living with cancer who consume cannabis, and factors associated with knowledge of cannabinoid concentrations.

**Methods:**

People living with cancer who consumed cannabis since their diagnosis (*n* = 343) completed an anonymous, mixed-mode survey. Questions assessed usual mode of delivery (MOD), knowledge of THC/CBD concentration, and how source of acquisition, current cannabis use, and source of instruction are associated with knowledge of THC/CBD concentration. Chi-square and separate binary logistic regression analyses were examined and weighted to reflect the Roswell Park patient population.

**Results:**

Less than 20% of people living with cancer had knowledge of THC and CBD concentration for the cannabis products they consumed across all MOD (smoking- combustible products, vaping- vaporized products (e-cigarettes), edibles-eating or drinking it, and oral- taking by mouth (pills)). Source of acquisition (smoking-AOR:4.6, *p* < 0.01, vaping-AOR:5.8, *p* < 0.00, edibles-AOR:2.6, *p* < 0.04), current cannabis use (edibles-AOR:5.4, *p* < 0.01, vaping-AOR: 11.2, *p* < 0.00, and oral-AOR:9.3, *p* < 0.00), and source of instruction (vaping only AOR:4.2, *p* < 0.05) were found to be variables associated with higher knowledge of THC concentration.

**Conclusion:**

Self-reported knowledge of THC and CBD concentration statistically differed according to MOD, source of acquisition, source of instruction, and current cannabis use.

## Introduction

In the USA, the use of medical cannabis is currently legal in 39 states and non-medical (recreational) use is legal in 23 states and the District of Columbia [1]. With increasing legalization across states, the cannabis market has been growing over the last decade, leading to diversification in the number of products that are available to consumers of both medical and non-medical cannabis [2, 3]. Products and modes of delivery that are currently available to consumers include smoking dried cannabis flower through joints, bongs, and pipes; vaping dried cannabis flower or oils; ingesting cannabis-infused food or beverages (edibles); ingesting capsules/tinctures; and topical formulations (e.g., cremes, salves).

The cannabis market is not only diverse in product type, but there is also great variation in cannabinoid content among products [4]. Cannabinoid content could potentially play an important role in determining the therapeutic efficacy or potential harms of these products among people living with cancer. There are over 100 known cannabinoids that have been isolated from the cannabis plant [5]. Two of the most prevalent cannabinoids are tetrahydrocannabinol (THC) and cannabidiol (CBD). THC is well known for its intoxicating effects. Its use can lead to memory impairment, paranoia, and potential for abuse, as well as longer-term outcomes such as increased risk of psychosis [6]. In addition to these risks, THC has been shown to have therapeutic effects. The studied benefits of THC include pain management, treatment of chemotherapy induced-nausea and vomiting, and improvement of multiple sclerosis spasticity [7]. CBD differs from THC in that it does not produce intoxicating effects [7–10]. CBD has been shown to have anti-inflammatory effects, and a commercially available pharmaceutical derived from CBD, (Epidiolex), is marketed and approved for treating rare forms of intractable epilepsy in children [11–13]. Not only are there differences in the effects of cannabinoids, there is significant variation in THC and CBD content within and across different cannabis products [14]. THC content of dried cannabis flower can range from < 1% to more than 30% [3, 14, 15], while concentrates can have THC content of upwards of 90% [14]. Edibles can vary from 5 to 7000 mg of THC per product and differ based on individual serving size or amount of THC in the entire package [16–18]. CBD-only products make up about 5% of the US cannabis market [19], with most products ranging from < 1% CBD up to 45% within a CBD-only product [20]. Notably, most cannabis products contain THC and CBD, with many of these products having high THC and low CBD ratios [19, 20]. Different ratios of THC:CBD can have varying individual and clinically relevant effects. Lower THC:CBD (1:2) ratios have been shown to attenuate negative effects of THC in healthy adults and adolescents, while higher THC:CBD (2:1) ratios can enhance the negative intoxicating properties of THC [6, 20]. In a recent experimental design, it was discovered that a low THC to high CBD dose of edible products (20 mg:640 mg, THC to CBD respectively) had the greatest ratings for feeling the negative effects of THC, including symptoms such as anxiousness, memory impairment, dry mouth, red or irritated eyes, and sickness, compared to THC alone [21]. Certain amounts of THC:CBD ratios have greater risks associated with their use as compared to benefits, and knowing if people living with cancer are aware of their products concentrations can help to shape discussions with providers about cannabis use.

Due to an increasing body of evidence that cannabis has therapeutic applications [13, 22–29], and the increasing availability of products, the use of cannabis has become more frequent among people living with cancer [22, 30, 31]. Previous survey research on the use of cannabis among those living with cancer has shown ranges from 18 to 21% of specific cancer patient populations using cannabis within the last 6 months [30, 32]. Among patients with cancer who consume cannabis, many consume to alleviate side effects of the disease and/or their cancer treatment [26, 33, 34], including pain, nausea, vomiting, loss of appetite, loss of sleep, anxiety, and depression [35–39]. Despite this, many providers typically lack knowledge on how and what to discuss with their patients when it comes to use of cannabis [40]. Given the variety of potential risks and benefits that various cannabis products can produce, it is essential for both oncologists and primary care providers to have access to information to assess the risks and benefits more appropriately for their patients. Higher levels of THC content have shown to increase the rate at which an individual could develop cannabis use disorder (CUD), further showing the need for why providers need to know about the cannabinoid levels in the products their patients are consuming [41]. The majority of people who consume nonmedical cannabis products regularly are not aware of the differences of concentration ratios (such as high THC or high CBD concentration) of the products that they use [2, 42, 43]. Factors such as legalization of medical and nonmedical cannabis in an individual’s state, and recency and frequency of cannabis use, have been shown to be associated with concentration knowledge [2] [43]. Additionally, jurisdictions that have legalized cannabis have regulations that cap maximum THC levels and require THC and CBD levels to be listed on products sold through legal channels, while concentration levels on products obtained from informal channels can be inconsistently labeled or not present [44, 45]. Degree of patient education on cannabis product characteristics and use may also be important to consider in evaluating whether patients are aware of concentration levels in the products they consume [46].

The main objective of this study was to evaluate self-reported knowledge of THC or CBD concentration in cannabis products used by individuals receiving cancer treatment and among those in cancer survivorship following the receipt of a cancer diagnosis at an NCI-designated Comprehensive Cancer Center located in New York, where cannabis was recently legalized for nonmedical use. We sought to address the following research questions: 1) Are patients aware of the amount of THC or CBD concentration in the products that they usually consume?; 2) Does self-reported knowledge of THC or CBD concentration differ by recency of use and usual mode of cannabis delivery?; 3) Are variables that may impact consumer knowledge of cannabis, such as source of instruction on how to use the product, source of product acquisition, and level of education, associated with self-reported knowledge of concentration in products? Given the above literature, we hypothesized that 1) overall self-reporting of THC and CBD concentration among patients who consume cannabis would be low; 2) self-reported knowledge of concentration would vary according to usual mode of delivery due to a diverse marketplace for cannabis products; and 3) self-reported knowledge of concentration would vary based on usual sources on instruction on cannabis (informal sources such as friends versus formal sources such as health providers), usual place of purchase (legal versus nonlegal markets), and level of patient education.

## Materials and methods

### Data source and methods

This study uses cross-sectional data from a mixed-mode survey distributed to patients and survivors at Roswell Park Comprehensive Cancer Center in Western New York from November 2021–May 2022. A sampling frame of potentially eligible participants was constructed using billing data prior to the start of the study. From this frame, a random sample of 10,000 adult (18 +) cancer patients undergoing active treatment and individuals who had completed their cancer treatment within the past year were invited to participate in a one-time, anonymous mixed mode survey that consisted of mail, web and telephone delivery methods. Participants were also required to reside in one of the eight counties within the immediate Roswell Park eight-county catchment area (Erie, Niagara, Genesee, Allegany, Orleans, Wyoming, Cattaraugus, Chautauqua). Recruitment efforts led to a total of 785 surveys collected. The initial mailing and online recruitment efforts collected 526 of those surveys, then 152 from a portal email, 96 by phone, and lastly 11 were collected through standard mail. All surveys were conducted in English, and respondents provided consent prior to beginning the survey. Those who participated in the survey were offered the chance to enter a $100 gift card drawing as an incentive. The overall response rate was 8%. The study was reviewed and approved by the Roswell Park Institutional Review Board.

### Measures

## Key outcomes

To understand concentration knowledge, we asked: “Please select whether you know the (THC or CBD) percentage (%) or a number of milligrams (mg). If you are not sure, please select not sure.” This variable was coded as categorical variables (one for THC, one for CBD) each consisting of three response options: 1) reported concentration (THC or CBD) in %, 2) reported concentration (THC or CBD) in milligrams (mg), and 3) not sure THC or CBD concentration. These variables were further collapsed to represent knowledge of concentration (either in % or mg) versus no concentration knowledge.

### Key exposures: mode of delivery and/or formulation

Consumers were asked to report the different modes in which they consumed cannabis following their cancer diagnosis from a select all that apply list. Modes that were available for selection included 1) smoking such as in a joint, bong, pipe, or blunt, 2) eating it in food such as brownies, cakes, cookies, or candy, 3) drinking it in a liquid such as tea, cola, or alcohol, 4) taking by mouth such as pills, tinctures, or sublingually (under the tongue), 5) vaping or vaporizing such as in an e-cigarette-like vaporizer or other vaping device, 6) dabbing using hot plate or dab rig to inhale shatter, wax, or butter, 7) applying topically in the form of a lotion, cream, or patch, and 8) other. These categories were collapsed into the following four modes of delivery to examine the association between knowledge of THC or CBD:

1. Smoking (joint, bong, pipe, or blunt),
2. Vaping (vaporizing such as in an e-cigarette-like vaporizer or other vaping device),
3. Edibles (eating it in food such as brownies, cakes, cookies, or candy, and drinking it in a liquid such as tea, cola, or alcohol)
4. Oral (taking by mouth such as pills, tinctures, or sublingually).

Each group contains individuals who selected yes to using that product. Four separate categories were used to assess the understanding of concentration knowledge of THC and CBD products due to the variation of labelling practices in the nonmedical cannabis marketplace.

## Source of acquisition

Cannabis acquisition source was assessed by asking respondents, “*Where do you typically get your cannabis*?” (response options included 1) I grow it myself, 2) I purchase it on the internet, 3) I get it from a friend or family member, 4) I get it from an unlicensed cannabis dealer or seller, 5) I get it from a medical dispensary by certification, 6) I get it from a recreational cannabis store/dispensary in another state/country, and 7) other”). Responses were recoded to represent: 1) Formal acquisition source (I get it from a medical dispensary by certification; I get it from a non-medical cannabis store/dispensary in another state/country), or 2) Informal acquisition source (I grow it myself; I purchase it on the internet; I get it from a friend or family member; I get it from an unlicensed cannabis dealer or seller).

## Source of instruction

Cannabis use instruction source was assessed through the question, “Who is the main person that gives you instructions on how to use cannabis and how much to take?” Response options included the following: 1) Primary care provider, 2) oncologist involved with your cancer treatment, 3) nurse or physician’s assistant involved with your cancer treatment, 4) pharmacist, nutritionist or dietician, 5) cannabis store or dispensary worker, 6) unlicensed cannabis dealer or seller, 7) another cancer patient, 8) friend or family member, 9) myself, and 10) other. These were recoded into: 1) Formal instruction source (Primary care provider; oncologist involved with your cancer treatment; nurse or physician’s assistant involved with your cancer treatment; pharmacist; nutritionist or dietician; and cannabis store or dispensary worker), and 2) Informal instruction source (unlicensed cannabis dealer or seller; another cancer patient; friend or family member; and patient’s own instruction). The “other” response options were coded by a member of the research team. Most “other” response options included themes related to original response categories. Many participants listed other healthcare providers such as neurologists, pharmacists, or names of legal dispensaries which were sorted as formal sources of instruction. Some participants listed themselves or a friend being their source of instruction, which was coded as informal.

## Recency of cannabis use

Current cannabis use was assessed by asking, “Are you currently using cannabis?” (Binary yes/no response option) among those who reported consuming cannabis since their diagnosis. Those who selected yes are considered users who are actively consuming cannabis.

## Other covariates

Sociodemographic covariates included age (18–34 years old, 35–49 years old, and 50 years or older); gender identity (male, female, and other (transgender & nonbinary)); race/ethnicity (White (non-Hispanic), Black (non-Hispanic), Hispanic, and all other); education (high school or less, vocational training/some college, bachelor’s degree, and postgraduate); and income (less than $35,000, $35,000–$74,999, $75,000–$99,999, equal to or more than $100,000, and refused). Education was recategorized as low (high school or less), medium (vocational training or some college), and high education (college graduate and higher) due to small cell counts in some categories (less than 50). Cancer stage was collected from the survey question “What stage was your cancer at the time you were diagnosed?” Response options included stage 0, stage 1, stage 2, stage 3, and stage 4. Those who did not answer were classified as missing. Much of our sample had similar cancer types (mostly head and esophageal cancers) therefore cancer type was not considered in our analyses. Reasons for cannabis use assessed the purpose of use for each individual patient, whether that be medical or nonmedical use. The question asked “What were your reasons for using cannabis after your cancer diagnosis? Select all that apply.” and response options included pain, mood changes, neuropathy, difficulty sleeping, difficulty concentrating, skin problems, sweating symptoms, digestive problems, lack of appetite, lack of energy or fatigue, lack of sexual interest of activity, used as a treatment or cure for cancer, used recreationally or for enjoyment, used for a cancer symptom or cancer treatment side effect not listed here, and other*.* Response options were recategorized as *recreational* (only checked used recreationally or for enjoyment), *medical* (checked any of the therapeutic indications and did not check recreational or for enjoyment), and *both* (patient checked both recreational and a therapeutic reason).

### Statistical analyses

All analyses were weighted to ensure findings were reflective of Roswell Park patients and survivors. Univariate analyses were utilized to describe the study population and key variables of interest. Chi-square tests were used to test for associations between key exposure and outcome variables and Cramer’s *V* was calculated to examine the effect size between exposure and outcome variables. No statistical associations were found between knowledge of cannabis concentration and age, race, gender, or income; therefore, these covariates were not included in regression models. Separate binary logistic regression models (restricted to consumers of the four most prevalent modes of cannabis delivery (smoking, edibles, vaping, and oral) were performed to test whether hypothesized factors were associated with self-reported knowledge of THC or CBD concentration (knows concentration versus does not know, modelled separately for THC and CBD, respectively). Each model examined associations with source of acquisition, source of instruction, current cannabis use, and educational attainment. All binary logistic regression models were weighted to reflect the Roswell Park patient population. Significance levels were set at *p* < 0.05, and all analyses were performed using SAS® software [47].

## Results

### Sample characteristics

Data from 343 cancer patients and survivors who self-reported cannabis consumption since cancer diagnosis were analyzed. Table 1 describes the weighted sample characteristics of cancer patients and survivors who have consumed cannabis since receiving a cancer diagnosis. Prevalence of cannabis use following cancer diagnosis was 26.5% (20.9% among patients in active treatment; 29.2% among survivors). The majority of our sample was 50 + years old (77.4%), white non-Hispanic (81.7%), and reported vocational training or some college (40.7%) as their highest form of educational attainment. Medical (62.9%) reasons were the most prominent reason for using a cannabis product, and the majority of patients who reported using cannabis after their cancer diagnosis were still currently using cannabis (63.6%) at the time of the survey. Poly-product cannabis use is common among consumers, although it was not found to be associated with our outcome of interest. This led to the creation of non-mutually exclusive models that are outlined in the following results for different modes of cannabis administration.

**Table 1:** Weighted sample characteristics (*n* = 343)

Age	*N*	Weighted %
*18–34 years old*	68	7.3%
*35–49 years old*	48	15.3%
*50 years or older*	227	77.4%
Gender Identity		
*Male*	146	43.5%
*Female*	192	55.8%
*Other (Transgender & Nonbinary)*	5	0.7%
Race		
*White, non-Hispanic*	297	81.7%
*Black, non-Hispanic*	20	6.6%
*Hispanic*	14	2.1%
*Other*	12	9.5%
Education		
*High school or less*	65	20.0%
*Vocational training/Some college*	144	40.7%
*Bachelor's degree*	89	27.4%
*Postgraduate*	45	11.9%
Income		
*Less than $35,000*	77	19.2%
*$35,000-$74,999*	86	24.9%
*$75,000-$99,999*	51	14.1%
*Equal to or more than $100,000*	81	25.9%
*Refused*	48	15.9%
Current Cannabis Use		
*Currently using*	241	63.6%
*Not currently using*	100	35.7%
*Missing*	2	0.6%
Reason for use		
*Medical*	197	62.9%
*Recreational*	27	8.3%
*Both*	119	28.9%
Cancer Stage		
*Stage 0*	43	12.4%
*Stage I*	88	24.4%
*Stage II*	60	16.5%
*Stage III*	60	15.5%
*Stage IV*	60	19.5%
*Missing*	32	11.7%

### Concentration reporting among consumers

**Fig. 1:**
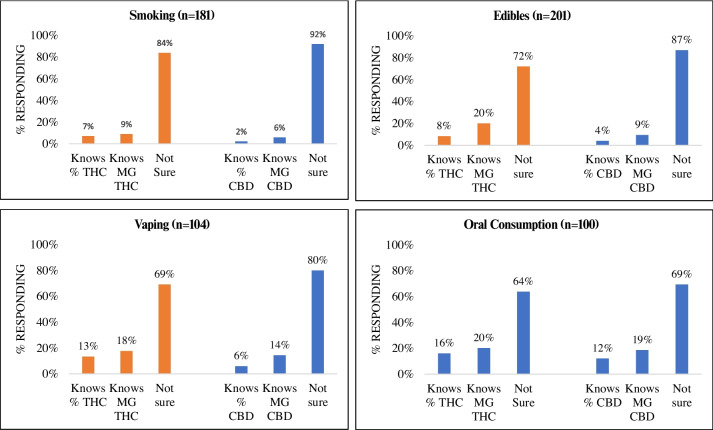
Self-reported knowledge of CBD and THC levels in cannabis products, according to mode of administration

Figure 1 shows the self-reported knowledge of THC and CBD concentration by mode of administration among patients who consumed cannabis since their cancer diagnosis. Dabbing and topical modes of delivery were excluded from this analysis due to small cell sizes (1 and 18, respectively). The lowest rates of cannabinoid concentration knowledge were among those who smoke cannabis (16.1% for THC and 8.1% for CBD), followed by those who eat cannabis foods or drinks in the edibles group (28.1% for THC and 13.2% for CBD). Slightly more self-reported cannabinoid knowledge was present among those who vaped (30.9% for THC and 20.1% for CBD), and those with the highest rates of self-reported cannabinoid concentration knowledge were those who consume products orally such as pills, tinctures, or sublingually (36.2% for THC and 30.7% for CBD).

### Variables associated with consumer knowledge of concentration

Table 2 shows the results from the chi-square tests of association across all products. Current cannabis use was significantly associated with self-reported knowledge of THC concentration (Χ^2^(2) = 21.5, Cramer’s *V* = 0.26, *p* < 0.001) and self-reported knowledge of CBD concentration (Χ^2^(2) = 9.2, Cramer’s *V* = 0.17, *p* < 0.01). For source of acquisition and source of instruction, there was a significant relationship for both self-reported knowledge of THC concentration (Χ^2^(2) = 33.2, Cramer’s V:0.32, *p* < 0.00 & Χ^2^(2) = 22.7, Cramer’s V:0.27, *p* < 0.00) and self-reported knowledge of CBD (Χ^2^(2) = 42.7, Cramer’s V:0.37, *p* < 0.00, and Χ^2^(2) = 35.0, Cramer’s V:0.33, *p* < 0.00) concentration.

**Table 2:** Variables associated with self-reported knowledge of CBD and THC concentration in cannabis products used since cancer diagnosis (*n* = 343)

Variable Assessed	Knows % CBD	Knows MG CBD	Not Sure	X^2^ Value (Cramer’s V)	*p*-value	Knows % THC	Knows MG THC	Not Sure	X^2^ Value (Cramer’s V)	*p*-value
Current Cannabis Use*										
*Currently Using (n* = *241, 64%)*	4.6%	9.0%	50.4%	9.2 (0.17)	*p* < 0.01	7.7%	13.7%	42.6%	21.5 (0.26)	*p* < .0001
*Not Currently Using (n* = *100, 36%)*	0.8%	2.2%	33.0%			0.6%	3.2%	32.1%		
Source of Acquisition_1_										
*Informal (n* = *251, 72%)*	1.0%	5.0%	65.5%	42.7 (0.37)	*p* < .0001	2.9%	9.1%	59.6%	33.2 (0.32)	*p* < .0001
*Formal (n* = *92, 28%)*	4.4%	6.1%	17.9%			5.3%	7.8%	15.3%		
Source of Instruction*_2_										
*Informal (n* = *244, 70%)*	1.3%	4.9%	64.1%	35.0 (0.33)	*p* < .0001	4.3%	8.2%	57.8%	22.7 (0.27)	*p* < .0001
*Formal (n* = *97, 30%)*	4.1%	6.3%	29.7%			4.0%	8.8%	16.8%		
Education_3_										
*Low (n* = *65, 20%)*	1.2%	1.8%	16.9%	1.7 (0.05)	*p* < .86	1.2%	1.7%	17.0%	12.5 (0.14)	*p* < .06
*Med (n* = *144, 41%)*	2.3%	3.9%	34.5%			3.8%	5.1%	31.8%		
*High (n* = *134, 39%)*	1.8%	5.4%	32.1%			3.3%	10.0%	26.0%		

^*^Missing 2

*a)* Cannabis Acquisition Source: Informal (unlicensed seller), Formal (licensed seller)

*b)* Cannabis Use Instruction Source: Informal (personal such as friends, family, and self), Formal (healthcare professional, such as nurse, physician, etc.)

*c)* Education Status: Low (high school or less), Med (vocational training or some college), and high (bachelors or higher)

Table 3 outlines results from regression models that examined self-reported knowledge of THC concentration according to usual mode of delivery. Among those who smoked cannabis, source of acquisition (AOR: 4.6, 95% CI 1.5–13.7, *p* < 0.01) and only high educational attainment (AOR: 3.6, 95% CI 1.1–12.4, *p* < 0.04) were associated with knowledge of THC concentration. Among those who consume edibles, source of acquisition (AOR: 2.6, 95% CI 1.0–6.6, *p* < 0.04), current cannabis use (AOR: 5.4, 95% CI 1.6–17.8, *p* < 0.01), and only high educational attainment (AOR: 4.0, 95% CI 1.0–16.6, *p* < 0.05), were associated with knowledge of THC concentration. Among those who vaped, source of acquisition (AOR: 5.8, 95% CI 1.9–18.1, *p* < 0.00), current cannabis use (AOR: 11.2, 95% CI 2.7–46.4, *p* < 0.00), and only high educational attainment (AOR 8.2, 95% CI 1.7–39.6, *p* < 0.01), were associated with knowledge of THC concentration. Lastly, among those in the oral group, source of instruction (AOR: 4.2, 95% CI 1.0–17.8, *p* < 0.05), and current cannabis use (AOR 9.3, 95% CI 2.4–36.0, *p* < 0.00), were associated with knowledge of THC concentration.

**Table 3:** Self-reported knowledge of THC concentration by mode of delivery selected by individuals consuming cannabis since cancer diagnosis

	Smoking (*n* = 181)	Edibles (*n* = 201)	Vaping (*n* = 104)	Oral (*n* = 100)
AOR (95% Confidence Intervals)				
Current Cannabis Use				
*Currently Using*	4.5 (0.8–26.0)	**5.4 (1.6–17.8)**	**11.2 (2.7–46.4)**	**9.3 (2.4–36.0)**
*Not Currently Using*	Ref	Ref	Ref	Ref
Source of Acquisition				
*Informal*	Ref	Ref	Ref	Ref
*Formal*	**4.6 (1.5–13.7)**	**2.6 (1.0–6.6)**	**5.8 (1.9–18.1)**	1.5 (0.4–5.9)
Source of Instruction				
*Informal*	Ref	Ref	Ref	Ref
*Formal*	0.8 (0.2–3.3)	2.6 (1.0–7.4)	0.7 (0.2–2.4)	**4.2 (1.0–17.8)**
Education				
*Low*	Ref	Ref	Ref	Ref
*Med*	0.8 (0.2–3.2)	1.4 (0.4–5.7)	1.2 (0.3–5.8)	0.8 (0.1–4.4)
*High*	**3.6 (1.1–12.4)**	**4.0 (1.0–16.6)**	**8.2 (1.7–39.6)**	4.0 (0.7–21.7)

a) Significant differences are indicated in bold (*p *< 0.05)

b) Four separate binary logistic regression models were run, one for each mode of delivery (smoking, edibles, vaping, and oral). Each model controlled for current cannabis use, source of acquisition, source of instruction and education status

Table 4 outlines results from regression models that examined self-reported knowledge of CBD concentration according to usual mode of delivery. Among those who smoke, source of acquisition (AOR: 6.5, 95% CI: 1.8–22.5, *p* < 0.00) was associated with knowledge of CBD concentration. Among those who consumed edibles, source of acquisition (AOR: 3.5, 95% CI 1.2–10.5, *p* < 0.02), and source of instruction (AOR: 3.8, 95% CI 1.3–11.0, *p* < 0.01) was associated with knowledge of CBD concentration. Among those who vaped, only source of acquisition (AOR: 5.1, 95% CI 1.4–18.3, *p* < 0.01) was associated with knowledge of CBD concentration. Lastly, among the oral group, only current cannabis use was associated with knowledge of the CBD concentration (AOR: 4.8, 95% CI 1.3–17.2, *p* < 0.02). Educational attainment did not show associations for the self-reported knowledge of CBD concentration (Χ^2^ = 1.7(4), Cramer’s V:0.05, *p* = 0.86).

**Table 4:** Self-reported knowledge of CBD concentration by mode of delivery selected by individuals consuming cannabis since cancer diagnosis

	Smoking (*n* = 181)	Edibles (*n* = 201)	Vaping (*n* = 104)	Oral (*n* = 100)
AOR (95% Confidence Intervals)				
Current Cannabis Use				
*Currently Using*	1.8 (0.4–8.2)	2.4 (0.6–9.7)	3.0 (0.6–16.0)	**4.8 (1.3–17.2)**
*Not Currently Using*	Ref	Ref	Ref	Ref
Source of Acquisition				
*Informal*	Ref	Ref	Ref	Ref
*Formal*	**6.5 (1.8–22.5)**	**3.5 (1.2–10.5)**	**5.1 (1.4–18.3)**	2.8 (0.7–10.2)
Source of Instruction				
*Informal*	Ref	Ref	Ref	Ref
*Formal*	0.6 (0.09–3.7)	**3.8 (1.3–11.0)**	1.7 (0.5–6.3)	1.9 (0.5–7.2)
Education				
*Low*	Ref	Ref	Ref	Ref
*Med*	0.5 (0.1–2.4)	0.7 (0.2–3.1)	2.0 (0.3–16.1)	0.6 (0.1–3.2)
*High*	0.8 (0.2–3.2)	0.6 (0.1–2.6)	2.5 (0.3–21.0)	1.5 (0.3–6.9)

a) Significant differences are indicated in bold (*p *< 0.05)

b) Four separate binary logistic regression models were run, one for each mode of delivery (smoking, edibles, vaping, and oral). Each model controlled for current cannabis use, source of acquisition, source of instruction and education status

## Discussion

This study sought to uncover if cancer patients and survivors were aware of the THC or CBD concentration in the products that they usually consumed following their diagnosis. The lowest levels of knowledge of concentration for both THC and CBD were observed for those who smoked cannabis, while knowledge levels were higher for more novel products (edibles, vapes). Several variables were analyzed to see if there was an association between knowledge of THC or CBD concentration and use. Source of acquisition, source of instruction, and current cannabis use were found to be associated with greater knowledge of THC and CBD concentration. Our core findings echo results from studies in the published literature conducted in non-clinical populations [42, 43, 48], and have important implications for future research and clinical practice.

Our findings showed that across all modes of delivery, only a low percentage of cancer patients and survivors who consumed cannabis since cancer diagnosis reported the THC or CBD concentration amount. This is consistent with the literature that many current cannabis consumers have low levels of knowledge related to the concentration of the products that they are using [2, 43]. The mode of delivery is an important consideration with regard to knowledge of cannabinoid concentration since variation in concentration may be tied to the pharmacokinetic properties and potential benefits or risks of these products [49–52]. Highly THC concentrated products have been linked to development of cannabis use disorder [41], and naïve users need to be educated on the risks of these products. Furthermore, those who had higher levels of education were more likely to know the THC concentration as compared to those in lower educational attainment categories. Those with higher levels of education may be able to more easily interpret the differences in percentages and milligrams, giving them more knowledge of the THC concentration. These factors should be considered to help inform labelling practices, and aid with the development of education for all consumers. Mass media education for consumers and training for healthcare providers should be considered to improve knowledge of cannabinoid concentration and its role in both the risks and benefits of use.

Source of acquisition was associated with knowledge of THC concentration in multiple modes of delivery including smoking, edible consumption, and vaping. Additionally, source of acquisition was found to be associated with knowledge of CBD products among individuals who vaped. To our knowledge, no other studies have assessed source of acquisition as a variable that is associated with knowledge of THC or CBD concentration among the products that they are consuming. Navigating the approval process for medical authorization, higher costs, and lack of access to certain products were noted as the main reasons for patients to continue to use informal sources to acquire their cannabis post-legalization [15, 53, 54]. Understanding where cannabis users of both medical and nonmedical cannabis purchase their products is vital due to the regulatory processes that are required of legal sources [20, 55]. In addition, source of instruction was found to be associated with knowledge of THC concentration among those who vape, and knowledge of CBD concentration among those who consume edibles. Currently there are no established guidelines for an efficacious dose of THC, CBD, or a combination of both for management of various cancer symptoms [42, 43, 48]. Cancer patients and survivors have noted that they have discussed their use of cannabis for medical purposes with providers, but did not receive formal advice on how to use the products [31, 40, 56]. More research is needed to understand how source of acquisition and source of instruction play a role in consumers’ understanding of the products that they are using.

In addition to exploring the associations of knowledge of THC and CBD concentrations with source of instruction and source of acquisition, dose interpretation could contribute to low-levels of understanding. Other studies that have looked at dosing interpretation have discovered that many patients are misinterpreting the correct dosing amount [57, 58]. With the variation of products available to consumers, the THC or CBD concentration specified dose per package can vary greatly. Those with a lower level of numeracy understanding may have difficulties interpreting the package labelling for these products. Lower literacy rates were cited as a reason for contributing to these misinterpretations. In a study focused on diabetes health literacy, it was discovered that beliefs about the medication were directly tied to their health literacy. Furthermore, numeracy literacy acted as a moderator on their illness perception and the medication adherence [59]. Future research should focus on understanding health and numeracy literacy among cancer patients who use cannabis.

The majority of cancer patients and survivors who are currently consuming cannabis from this study do not know the THC or CBD concentration of the products that they are consuming. This study implies that providers should be cautious when relying solely on patient self-reported data for an accurate measurement of THC or CBD concentration levels. This study brings to light some of the factors that are associated with increased knowledge of cannabinoid levels. Source of acquisition and source of instruction were found to have small associations with knowledge of concentration, suggesting that there is a need for more formal education among people living with cancer to improve knowledge of THC or CBD concentration.

### Strengths and limitations

To our understanding, this is the first study to look at knowledge of THC and CBD concentration in a sample of cancer patients and survivors. This study is also one of the first to test for variables associated with concentration knowledge, including source of acquisition and source of instruction. The non-exclusivity of our groups for modes of delivery gave us greater insights into how different types of products are associated with knowledge of THC or CBD concentration. Our primary finding of low levels of knowledge of THC or CBD in products most frequently used by consumers also aligns with similar studies conducted in the general population [42, 43].

A limitation of this study was that our data is not nationally representative and can only give insights into a cancer patient population located in the Western New York region. Our sample was reflective of the Roswell Park patient population, which consists of mostly white non-Hispanic individuals. Future research should focus on collecting nationally representative data on cancer patients who consume cannabis to better understand cannabis use patterns among different racial/ethnic minority groups. Another limitation is the 8% response rate for our study. By weighting our data to reflect the Roswell Park patient population, the study team was able to minimize any potential bias issues. Lastly, recency bias could have impacted the results of our survey. Our sample included individuals in different stages of their cancer treatment.

## Conclusion

Most cancer patients and survivors who consume cannabis are unaware of the THC or CBD concentration in the products they usually consume. These data suggest that relying on patient self-report of cannabinoid levels is unlikely to provide useful or accurate information to providers. Future research should focus on increasing healthcare provider training and knowledge of cannabis and its features, to help improve patient knowledge to help maximize potential risks and benefits that may come from cannabis use during treatment and survivorship.

## Data availability statement

The data generated in this study are not publicly available due to information that could compromise patient privacy or consent. Specific data requests can be made by reaching out to Danielle Smith at Danielle.smith@roswellpark.org.

## Declarations

We confirm that we have read the Editorial Policy pages. This study was conducted with the approval from the Roswell Park Institutional Review Board, located in Buffalo, New York. This study was conducted in accordance with the Declaration of Helsinki. Written informed consent was obtained from all participants.
